# Prevention and potential remedies for antibiotic resistance: current research and future prospects

**DOI:** 10.3389/fmicb.2024.1455759

**Published:** 2024-10-03

**Authors:** Rabiya Tabbassum Khan, Vanshika Sharma, Sofia Sharief Khan, Shafaq Rasool

**Affiliations:** Molecular Biology Lab, School of Biotechnology, Shri Mata Vaishno Devi University, Katra, India

**Keywords:** antibiotics, antibiotic resistance, prevention strategies, glass, multidrug resistance, combinational therapy

## Abstract

The increasing threat of antibiotic resistance and shrinking treatment options for infections have pushed mankind into a difficult position. The looming threat of the return of the pre-antibiotic era has caused a sense of urgency to protect and conserve the potency of antibiotic therapy. One of the perverse effects of antibiotic resistance is the dissemination of its causative agents from non-clinically important strains to clinically important strains and vice versa. The popular saying “Prevention is better than cure” is appropriate for tackling antibiotic resistance. On the one hand, new and effective antibiotics are required; on the other hand, better measures for the use of antibiotics, along with increased awareness in the general public related to antibiotic use, are essential. Awareness, especially of appropriate antibiotic use, antibiotic resistance, its dissemination, and potential threats, can help greatly in controlling the use and abuse of antibiotics, and the containment of antibiotic resistance. Antibiotic drugs’ effectiveness can be enhanced by producing novel antibiotic analogs or adding adjuvants to current antibiotics. Combinatorial therapy of antibiotics has proven successful in treating multidrug-resistant (MDR) bacterial infections. This review aims to highlight the current global situation of antibiotic resistance and discuss the methods used to monitor, prevent, inhibit, or reverse bacterial resistance mechanisms in the fight against antibiotic resistance.

## 1 Introduction

**Figure 1:**
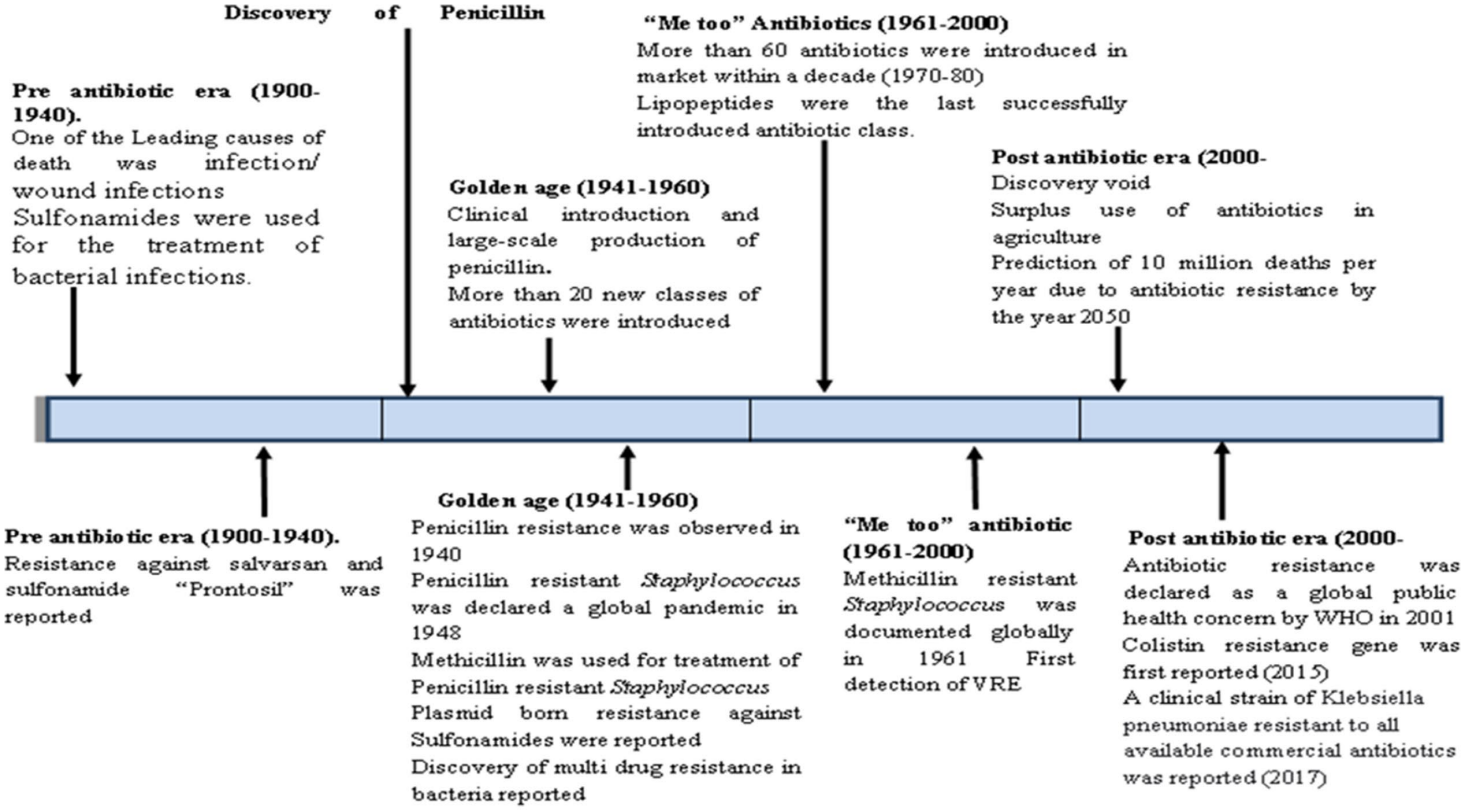
Timeline of discovery of antibiotics and antibiotic resistance.

Antibiotics are naturally occurring compounds or their derivatives produced by bacteria or fungi that can kill or inhibit other competing microbes. They can be simply defined as antimicrobial agents, either natural, synthetic, or semi-synthetic, that kill bacteria or prevent bacterial growth. Their discovery transformed the field of medicine in many aspects, as they have been crucial in the fight against various bacterial infections and diseases for a very long time. The period from 1950 to 1970 was the true “golden age,” as half of the antibiotics commonly used today were either discovered in this period or were reintroduced later with modifications (Kolter, 2021). Primarily, they have been utilized to treat bacterial infections in humans, but during the last few decades, they have been used extensively in various other fields like industry, agriculture, aquaculture, etc. As the use of antibiotics became prevalent among different day-to-day activities, the ability of bacteria to withstand these antibiotics also evolved. This ability of bacteria to resist the effects of antibiotics to which they have previously been sensitive is called *“antibiotic resistance*.” Antibiotic resistance is a natural phenomenon, as evidenced by self-resistance in antibiotic-producing microbes and the coexistence of antibiotic and non-antibiotic producers in the same niches. The observation of resistance against the antibiotic penicillin by the enzyme penicillinase even before it was developed as a therapeutic agent also shows that antibiotic resistance phenomenon is natural (Lobanovska and Pilla, 2017). Although bacterial resistance to certain antimicrobial compounds was discovered before antibiotics were developed, it was not until the widespread use of antibiotics that this issue gained significant attention (Figure 1). Antibiotic-resistance genes are widely prevalent in the environment and are not restricted to clinical microbes only (Peterson and Kaur, 2018). Though the resistance in bacterial communities in the environment does not directly threaten human welfare, their mobilization to new hosts and expression under different conditions can cause great damage. Selective pressure caused by human activities has enriched such elements/determinants in the bacterial population. The co-existence of antibiotic-producer and non-producer bacteria in the environment has also resulted in the evolution of antibiotic resistance mechanisms in non-producing bacteria. For example, *Streptomyces* species produce a variety of antibiotics, including streptomycin and tetracycline, and bacteria that do not produce antibiotics (non-producers) share the same environment with these antibiotic-producing strains (Peterson and Kaur, 2018; Martínez, 2018). Antibiotics work against bacteria by disrupting their physiological or biochemical pathways, like inhibition of cell wall synthesis by penicillin and other beta-lactam antibiotics. Beta-lactams bind to and inhibit penicillin binding proteins (PBPs), which are crucial for the cross-linking of peptidoglycan layers in the bacterial cell wall. Without a proper cell wall, the bacteria cannot maintain their structural integrity and die. Other drug targets include protein synthesis inhibition by macrolides and inhibition of essential enzymes such as DNA gyrase and topoisomerase IV by fluoroquinolones. To resist antibiotics, a microbe can adopt one or more than one of the following strategies (a) excretion of antibiotics by efflux; (b) modification of target of antibiotics; (c) alterations in permeability of outer membrane; and (d) inactivation or degradation of antibiotics by bacterial enzymes. The emergence of multidrug resistant (MDR), pan-drug resistant (PDR), or extremely drug-resistant (XDR) bacterial strains are also attributed to the presence of more than one resistance mechanism (Reygaert, 2018). MDR are defined as acquired nonsusceptibility to at least one agent in three or more antimicrobial categories, XDR are defined as nonsusceptibility to at least one agent in all but two or fewer antimicrobial categories and PDR are defined as nonsusceptibility to all agents in all antimicrobial categories.^1^

Some examples of antibiotic resistance mechanisms are shown in Table 1.

**Table 1:** Resistance mechanisms employed by different bacteria.

Mechanism of resistance	Resistant organism	Resistance against antibiotics	References
Alternation in membrane permeability	*Enterococci, S. aureus, Enterobacteriaceae, Enterobacter aerogenes*, *Neisseria gonorrhoeae*	Aminoglycosides, Vancomycin, Carbapenems, Imipenem and certain cephalosporins, β-lactams and Tetracycline	Kwon (2017), Reygaert (2018), Munita and Arias (2016), Philippe et al. (2015), Onishi et al. (2022), Gonzales Zamora and Camps (2018), Pang et al. (2019)
Modification of antibiotic’s target	*S. aureus*, *Enterococci, E. coil, M. tuberculosis, Streptococcus pneumoniae, S. enterica, Helicobacter pylori*	β-lactams, Methicillin, Vancomycin, Fluoroquinolone, Rifampicin, Macrolide, Chloramphenicol, Tetracycline	Li G. et al. (2022), Schaenzer and Wright (2020), Peterson and Kaur (2018), Luthra et al. (2018)
Active efflux pump	*P. aeruginosa, E. coli. N. gonorrhoeae, Burkholderia cepacia complex, Stenotrophomonas maltophilia, Haemophilus influenzae*	Fluoroquinolones, Tetracycline, Chloramphenicol, β-lactams, Fluoroquinolones, Trimethoprim	Koo (2015), Atac et al. (2018), Nishino et al. (2021)
Inactivation or degradation of antibiotics	*Enterobacteriaceae S. aureus Acinetobacter* spp.	β-lactams, Carbapenems	Reygaert (2018), Kapoor et al. (2017), Yang et al. (2019), Smith and Kendall (2022)
	*Pseudomonas* spp.	β-lactams, Aminoglycosides, Chloramphenicol	

Genes/Factors responsible for antimicrobial resistance in the environment have gained momentous attention due to their possible link with the emergence of resistance in pathogenic clinical isolates (Peterson and Kaur, 2018). The throng of antibiotic-resistant strains in those environments where the bacteria have not been exposed to antibiotics suggests that resistance genes can be resolutely retained by bacteria even in the absence of antibiotic selection (Urban-Chmiel et al., 2022). This review deals with the current scenario of antibiotic resistance and the approaches involved in monitoring, preventing, inhibiting, or reversing resistance mechanisms in bacteria to battle against antibiotic resistance.

## 2 Antibiotic resistance—a global perspective and its surveillance

Antibiotic resistance has become a global threat (Aljeldah, 2022). Rapidly increasing resistance in bacteria, especially pathogenic ones, has raised concerns regarding health care. According to the World Health Organization (WHO), antimicrobial resistance (AMR) is one of the top 10 global public threats faced by humanity presently (World Health Organization, 2021). Accumulation of new antibiotic resistance mechanisms and its spread among pathogenic bacteria have threatened our ability to successfully treat common infections and diseases (Mancuso et al., 2021). The development and spread of superbugs such as MDR, XDR, and PDR strains (*Klebsiella pneumoniae, Acinetobacter baumannii, Pseudomonas aeruginosa*, Methicillin-Resistant *Staphylococcus aureus* (MRSA)) globally is a significant concern, as they result in infections that are difficult to treat or, in some cases, untreatable with existing antibiotics (Basak et al., 2016). Reports such as 35,000 deaths per year alone in the European Union due to antibiotic resistance complications (ECDC, 2022), more than 38,000 deaths per year in Thailand due to antibiotic resistance (Sumpradit et al., 2012), death of 58,000 babies in one year in India due to antibiotic-resistant bacterial infections which are usually passed down from their mothers (Laxminarayan et al., 2013), shows the existing dire situation witnessed globally due to antibiotic resistance. AMR has serious global implications for human health, the economy, and security. In 2015, WHO approved a *“Global Action Plan (GAP-AMR)”* to ensure that the successful treatment and prevention of infectious diseases is continuous with effective and safe medicines (antibiotics and antimicrobials) so that they are accessible when needed. WHO also launched a surveillance system known as the *“Global Antimicrobial Resistance and Use Surveillance System (GLASS)*” on 22^nd^ October 2015 to monitor the development of antibiotic resistance and its impact on local, national, and global strategies. GLASS is a collaborative effort to standardize AMR surveillance globally and to support the objective of GAP-AMR. These Global Action Plans will provide a standardized approach for collecting, analyzing, interpreting, and sharing data and promote the surveillance system inclusive of epidemiological, clinical, and population-level data. The full GLASS report of 2021 which summarizes the data of 2020, available on the WHO website, *presents* the current global scenario of antibiotic resistance, and *emphasizes* the need to address this crucial issue. Along with WHO GAP plan, many countries have developed approaches at regional levels that are tailored to local challenges and healthcare systems. These regional approaches to AMR often reflect variations in antibiotic use, public awareness, healthcare infrastructure, and resistance patterns. For example, the European Centre for Disease Prevention and Control (ECDC) has been a leader in organizing coordinated efforts against AMR across the EU. The European Antibiotic Awareness Day (EAAD), held annually, is one such initiative that focuses on educating both healthcare professionals and the public about the risks of inappropriate antibiotic use. The European Antimicrobial Resistance Surveillance Network (EARS-Net) monitors AMR trends in European countries and provides crucial data to inform local and regional interventions. The EU Joint Action on AMR and Healthcare-Associated Infections (EU-JAMRAI) works on strengthening antibiotic stewardship and infection prevention programs across member states. The strong surveillance systems and early stewardship programs in countries like Sweden and Netherlands have reported some of the lowest antibiotic resistance rates in the EU. The high availability of over-the-counter antibiotics and their widespread use in agriculture, and densely populated urban areas in Asia Pacific countries like India, China Thailand presents a unique challenge in managing the use of antibiotics. As a result, several countries have launched region-specific campaigns to address the diverse factors contributing to AMR. For example, In India, the National Action Plan on Antimicrobial Resistance (2017–2021) has been formed which focuses on strengthening infection control, promoting antibiotic stewardship in hospitals, and raising public awareness. India’s public health campaigns also emphasize reducing the misuse of antibiotics in agriculture. While India has made progress in implementing policies, challenges remain in rural areas where antibiotic regulation is less strict. In many parts of sub-Saharan Africa, AMR poses a severe threat due to limited access to healthcare, weak healthcare infrastructure, and the misuse of antibiotics in both human and veterinary medicine. Despite these challenges, several African countries have made strides through targeted regional campaigns. For example, Kenya has implemented a National Action Plan on AMR focusing on public awareness, antibiotic stewardship, and improved surveillance. One successful initiative involved training community health workers in remote areas to educate patients on the proper use of antibiotics. A reduction in antibiotic misuse in areas targeted by educational programs has been observed. Additionally, surveillance efforts have helped identify local resistance trends, improving the targeting of interventions (World Health Organization, 2022). Pan American Health Organization (PAHO) spearheaded the regional initiatives against challenges with the overuse of antibiotics, particularly in agriculture in Latin American countries. For example, Chile launched its National AMR Strategy in collaboration with PAHO, focusing on regulating antibiotic use in agriculture and promoting hospital-based infection control measures (Fabre et al., 2022). Countries like Jordan and Saudi Arabia from the Middle East and North Africa region emphasize reducing antibiotic misuse in hospitals, community awareness, and improving surveillance in the national action plan (Haseeb et al., 2023). Regional approaches to AMR vary widely depending on healthcare systems, cultural factors, and local resistance patterns. The judicial and controlled use of existing antibiotics among exploring and developing strategic tools against antibiotic resistance is the need of the hour. Some of these strategies are discussed in this review.

## 3 Prevention strategies against antibiotic resistance mechanism

Increasing antibiotic resistance in microorganisms has resulted in ineffective treatments with time, and there is an urgent need to conserve the potency of antibiotics. It is important to enhance the present-day antibiotics by producing new antibiotics that could replace the earlier ones or by extending the life or efficacy of current antibiotics by blocking resistance pathways (Goff et al., 2017). The various strategical responses against antibiotic resistance like the use of various inhibitors, the use of membrane permeabilizers like antibiotic adjuvants, the use of nanoparticles, plasmid curing, genome studies, etc. are some of the measures for preserving the efficacy of antibiotics as discussed below.

### 3.1 Increasing the efficacy of antibiotics

**Figure 2:**
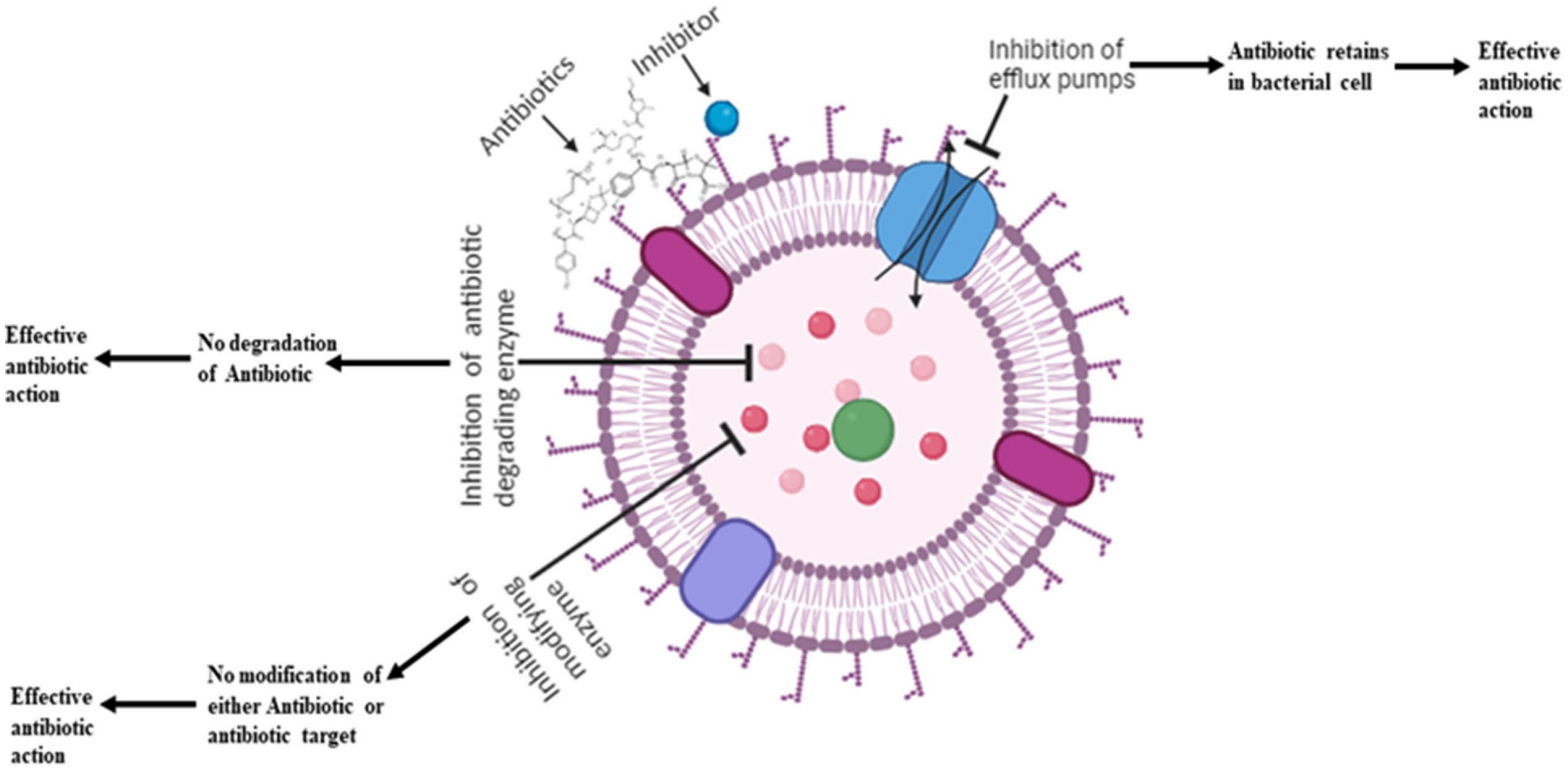
Various inhibitors used to increase the efficacy of antibiotics.

Recent studies have found that interactions and combinations of antibiotics with other antibiotics and compounds are a promising approach in the fight against resistance in bacteria, e.g., Amoxicillin in combination with clavulanic acid allows the inactivation of *β*-lactamase enzyme and allows amoxicillin to inhibit cell wall synthesis (Bush and Bradford, 2016). The use of various inhibitors, and membrane permeabilizers, like antibiotic adjuvants, nanoparticles, etc. are some of the approaches that can increase the efficacy of currently available antibiotics (Figure 2).

#### 3.1.1 Use of inhibitors in combination with antibiotics

The most common cause of antibiotic resistance in many clinically important bacteria includes biochemical mechanisms like efflux pumps and enzymatic resistance which cause inactivation or degradation of antibiotics. Efflux pumps are considered one of the main mechanisms of antibiotic resistance in both gram-positive and gram-negative pathogenic bacteria (Parrino et al., 2020). Bacterial enzymes also play a key role in the development of resistance. These enzymes participate in various biochemical pathways like modification of the antibiotic-targeted enzymes, modification of intracellular targets, transformation of antibiotics, and the implementation of cellular metabolism reactions resulting in ineffective antibiotic use. For example, Methyltransferases in *S. pneumoniae* and *S. aureus* methylate the 23S rRNA of the bacterial ribosome, altering the binding site for macrolides, lincosamides, and streptogramins (MLS antibiotics). This alternation results in resistance or VanA ligase synthesizes an altered cell wall precursor (D-Ala-D-Lac) that prevents vancomycin from binding effectively, leading to vancomycin resistance. Another example is the hydrolysis of the *β*-lactam ring of penicillins and cephalosporins by β-Lactamase enzymes, rendering the antibiotics ineffective. The main mechanisms of resistance development are associated with the evolution of super-families of bacterial enzymes which is due to the variability of the genes encoding them. Several enzymes can give rise to antibiotic resistance since they can hydrolyze the sensitive bonds within antibiotics, shift a functional group to the antibiotic, or facilitate redox reactions. Common examples of such enzymes are *β*-lactamases which act against *β*-lactam antibiotics, and aminoglycoside-modifying enzymes which act against the aminoglycoside class of antibiotics (Kapoor et al., 2017).

Beta-lactamases are enzymes produced by bacteria that hydrolyze the *β*-lactam ring of β-lactam rendering the antibiotic ineffective. They confer resistance to β-lactam antibiotics, including penicillins, cephalosporins, monobactams, and carbapenems. These enzymes are classified based on their molecular structure and catalytic mechanism. The Ambler classification is the most commonly used system, dividing *β*-lactamases into four classes A, B, C, and D based on amino acid sequence homology (Kaderabkova et al., 2022).

Class A *β*-lactamases are Serine β-lactamases. They are the most prevalent β-lactamases and include a variety of enzymes, such as penicillinases and extended-spectrum β-lactamases (ESBLs). These enzymes hydrolyze penicillins and cephalosporins. The active site contains a serine residue that plays a critical role in catalyzing the hydrolysis of the β-lactam ring. ESBLs are a subset of Class A *β*-lactamases that are capable of hydrolyzing extended-spectrum cephalosporins (e.g., cefotaxime, ceftazidime, ceftriaxone) and monobactams (e.g., aztreonam). ESBL-producing organisms are typically resistant to multiple β-lactam antibiotics, posing a significant challenge in clinical treatment. The key types of ESBLs include the TEM, SHV, and CTX-M families. The CTX-M type, in particular, has become widespread globally and is often associated with multidrug-resistant infections in healthcare and community settings. TEM ESBL family is named after the first patient, Temoneira, in which they were discovered. Till now more than 200 TEM ESBL have been recognized. TEM-1 is another prevalent *β*-lactamase, initially reported in *Escherichia coli*. TEM-1 hydrolyzes penicillins and early-generation cephalosporins. Mutations in the TEM gene have given rise to various ESBLs, such as TEM-3 and TEM-52, which have extended hydrolytic activity to include third-generation cephalosporins. SHV is the Sulfhydryl Variable ESBL. Originally, SHV enzymes were narrow-spectrum *β*-lactamases capable of hydrolyzing penicillins and early-generation cephalosporins. However, due to point mutations in the SHV gene, certain variants have evolved into ESBLs, which can hydrolyze a broader range of β-lactam antibiotics, including third-generation cephalosporins and monobactams. Both are the earliest and most well-known ESBLs. Mutations in these enzymes lead to a widened substrate spectrum, allowing them to hydrolyze third-generation cephalosporins. CTX-M are a relatively newer group of ESBLs and are becoming more prevalent. These enzymes have a strong affinity for cefotaxime and are increasingly associated with community-acquired infections (Philippon et al., 2016).

Class B *β*-Lactamases are Metallo-β-Lactamases (MBLs). They are metallo-enzymes that require divalent metal ions, usually zinc, for their catalytic activity. Unlike Class A, C, and D enzymes, which use a serine residue at their active site, Class B enzymes use a zinc ion to activate a water molecule, which in turn hydrolyzes the *β*-lactam ring. These enzymes have a broad-spectrum activity, including the ability to hydrolyze carbapenems—antibiotics often used as a last resort for treating multidrug-resistant bacterial infections. These enzymes are particularly concerning because they are not inhibited by traditional *β*-lactamase inhibitors, making infections difficult to treat. One of the examples is New Delhi Metallo-*β*-Lactamase NDM-1, that was first identified in a patient from New Delhi, which has spread globally and is found in various gram-negative bacteria, including *E. coli* and *K. pneumoniae*. It can hydrolyze all *β*-lactams except monobactams. VIM (Verona Integron-encoded Metallo-*β*-Lactamase) enzymes are widely distributed and found in *P. aeruginosa* and *Acinetobacter* species, contributing to their resistance to carbapenems. IMP (Imipenemase) that were originally discovered in *P. aeruginosa*. IMP enzymes can hydrolyze carbapenems and have been found in multiple bacterial species globally. MBLs are resistant to all *β*-lactamase inhibitors that target serine *β*-lactamases. However, they are inhibited by metal ion chelators like EDTA, although this is not clinically useful. The lack of effective inhibitors for MBLs represents a significant challenge in treating infections caused by these enzymes (Sawa et al., 2020; Tooke et al., 2019).

Class C *β*-Lactamases are also known as AmpC β-Lactamases. Class C β-lactamases are also serine-based enzymes with a similar catalytic mechanism to Class A enzymes. They hydrolyze *β*-lactam antibiotics by forming an acyl-enzyme intermediate, that is hydrolyzed to release the inactive antibiotic. They are primarily found in gram-negative bacteria species such as *Enterobacter, Citrobacter, Serratia,* and *Pseudomonas*. These enzymes hydrolyze a wide range of β-lactams, including penicillins, early-generation cephalosporins, and cephamycins (e.g., cefoxitin). AmpC *β*-lactamases can be either chromosomally encoded or plasmid-mediated. Chromosomal AmpC enzymes are typically inducible, meaning their expression can be upregulated in the presence of *β*-lactam antibiotics. Plasmid-mediated AmpC enzymes are constitutively expressed and can be transferred between bacterial species, contributing to the spread of resistance. These enzymes are generally resistant to inhibition by β-lactamase inhibitors such as clavulanic acid. This resistance complicates treatment, especially in cases where the bacterial strain also carries additional resistance mechanisms. Infections caused by AmpC-producing bacteria often require alternative antibiotics, such as carbapenems, although resistance to carbapenems is also emerging (Philippon et al., 2022).

Class D *β*-Lactamases are also known as OXA-type β-Lactamases because of their ability to hydrolyze oxacillin. They are serine-based enzymes with a unique structural fold. They catalyze the hydrolysis of *β*-lactam antibiotics through a mechanism similar to that of Class A enzymes, involving the formation of an acyl-enzyme intermediate. These enzymes are notable for their ability to hydrolyze oxacillin and cloxacillin, as well as some cephalosporins. More concerning, however, is their ability to hydrolyze carbapenems in some cases, making them significant contributors to antibiotic resistance in Gram-negative bacteria. Some examples of OXA-type *β*-Lactamases are OXA-48 which has a weak carbapenemase activity but does not hydrolyze extended-spectrum cephalosporins well. It is often found in *K. pneumoniae* and other *Enterobacteriaceae* and is a major cause of carbapenem resistance in these bacteria. OXA-23, OXA-24/40, and OXA-58 are prevalent in *A. baumannii* and contribute significantly to the carbapenem resistance. Class D *β*-lactamases, particularly those with carbapenemase activity, are resistant to many *β*-lactamase inhibitors. Some new inhibitors, such as avibactam, have shown activity against OXA-type enzymes, but the increasing diversity and spread of these enzymes continue to pose challenges in clinical settings (Yoon and Jeong, 2020).

The use of inhibitors against these enzymes will not only help in increasing the efficacy of the antibiotics but can also help in re-sensitizing the bacteria (González-Bello et al., 2020). Inhibitors of *β*-lactamases can be divided into two types: (a) inhibitors with a β-lactam core e. g. Clavulanic acid, Sulbactam, Tebipenem, etc., and (b) inhibitors without a β-lactam core, e.g., Avibactam, Relebactam (Luci et al., 2021). Some of the commonly used β-lactamases inhibitors (BLIs) are discussed below.

##### 3.1.1.1 Clavulanic acid

Clavulanic acid is a β-lactam drug that functions as a mechanism-based β-lactamase inhibitor. It was isolated from *Streptomyces clavuligerus*. It binds to the active site of β-lactamase via its β-lactam ring and obstructs enzymatic activity of the enzyme. It forms an inactive acyl-enzyme complex by acylation of catalytic serine residue of β-lactamases (mostly ESBLs and some Carbapenemases of Ambler Class A). It hampers the *β*-lactamases encoded by the plasmid of *E. coli* and *S. aureus*, but not the chromosomally encoded type present in the strains of *Enterobacter* and *Pseudomonas*. Similarly, a drug combination of amoxicillin and clavulanic acid is active against both amoxicillin-resistant and amoxicillin-sensitive strains. It is commercially available as Augmentin (clavulanate with amoxicillin/co-amoxiclav). Other examples include Timentin (clavulanate with ticarcillin/co-ticarclav) (Laws et al., 2019) and a clavulanate and piperacillin combination is available under brand name Tazocin or Zosyn (Harris et al., 2015). Other than these, combinations of clavulanate-aztreonam, clavulanate-ceftazidime, and clavulanate-aztreonam are being researched for their potential use in clinical settings (Livermore et al., 2018; Harris et al., 2015).

##### 3.1.1.2 Amino inhibitors

One of the current and major causes of *β*-lactam resistance is Metallo β-lactamases (MBLS). MBLs hydrolyze β-lactam antibiotics with the help of a divalent metal cofactor, typically zinc, unlike other β-lactamases. They encompass the full range of β-lactam antibiotics, including carbapenems, cephalosporins, and penicillins. MBLs can be inhibited by the naturally occurring poly amino acid aspergillomarasmine A (Tooke et al., 2019) The clinical usage of aspergillomarasmine A has reinstated meropenem’s activity. It is efficient against MBLs like NDM-1 and Verona Integron encoded Metallo β-lactamases (VIM-2) (Koteva et al., 2022). Although it shows potent activity in laboratory settings, it is not yet used in routine clinical practice. Another example of an amino inhibitor is a poly amino derivative of maleic acid that has potentialized ceftazidime and carbapenems is ME1071 which is quite effective against Imipenemase Metallo *β*-lactamases (IMP) (Palacios et al., 2020). While both aspergillomarasmine A and ME1071 represent promising advancements in the fight against MBL-mediated antibiotic resistance, they are not yet fully established in clinical practice. More research, clinical trials, and regulatory approvals are needed before these inhibitors can be used widely to combat infections caused by MBL-producing bacteria. For now, their usage remains primarily experimental. While these developments are promising, more research is needed to bring these inhibitors into routine clinical use, where they could significantly improve the treatment of infections caused by MBL-mediated antibiotic resistance.

##### 3.1.1.3 Diazabicyclooctane (DBO scaffold) inhibitors

Twenty years after the discovery of *β*-lactamase inhibitors, a class of non-β-lactam lactamase inhibitors was found based on diazabicyclooctane (DBO) scaffold. DBO scaffold has become the pillar of second-generation *β*-lactamase inhibitors. The first inhibitor of this class was Avibactam which acts as a covalent inhibitor in a reversible manner. It acts against Ambler class A, class C, and some class D *β*-lactamases. The opening of the avibactam ring leads to the covalent inhibition followed by the de-acylation of the covalent compound (acylated enzyme) resulting in the regeneration of the active inhibitor. Avibactam is used in combination with ceftazidime. Currently, other combinations of avibactam, such as ceftaroline-avibactam, and aztreonam-avibactam, which have shown *in vitro* potential of being effective against antibiotic-resistant bacteria are being developed (Li et al., 2015). Another DBO scaffold *β*-lactamase inhibitor is relebactam which is identical to avibactam, with piperazine ring at Carbon 2 amide substituent. It inhibits *β*-lactamases of carbapenemases, class A and class C (Sader et al., 2017), and is used in combination with imipenem and cilastatin. Zidebactam is also a DBO scaffold lactamase inhibitor that follows a dual mode of action (against PBP-2 and *β*-lactamases) like nacubactam. It acts straight against multi-drug resistant gram-negative bacteria like *P. aeruginosa* and *A. baumannii*. Zidebactam inhibits β-lactamases of class A, C, and some members of class D. It is used commercially in combination with cefepime and is going through successful phase I trials. Currently, many other diazabicyclooctanes are under development (Mallalieu et al., 2020).

##### 3.1.1.4 Boronic acid transition state inhibitors (BATSIs)

Another new class of inhibitors of *β*-lactamases is Boronic acid transition state inhibitors (BATSIs). It acts against serine β-lactamases and consists of boronic acid. Its electrophilic boron atoms mimic the electrophilic carbonyl center of the β-lactam ring and form an enzyme-BATSI adduct when attacked by the β-lactamase enzyme which leads to the inhibition of the enzyme in a reversible and competitive method of inhibition. Presently, vaborbactam is one of the highly efficient BATSI available commercially (Peppoloni et al., 2020; Powers et al., 2023). It inhibits the β-lactamases of class A, C, D, and KPC (*Klebsiella pneumoniae* carbapenemase enzyme produced by certain bacteria, particularly *K. pneumoniae*, that confers resistance to carbapenem antibiotics), CTX-M, SHV, and CMY (Lomovskaya et al., 2017; Hecker et al., 2015). VNRX-5133 (taniborbactam), which works by mimicking the substrate of β-lactamase enzymes, is another BATSI under clinical development (currently in phase 3 clinical trials). It is the first pan spectrum beta-lactamases inhibitor that inhibits the β-lactamases of class A, C, D, and also, VIM/NDM class B metallo β-lactamases (Liu et al., 2020). Several *in vitro* studies on the combination of VNRX-5133 with cefepime against carbapenem-resistant Enterobacteriaceae and *P. aeruginosa* have been reported (Kloezen et al., 2021; Hamrick et al., 2020; Krajnc et al., 2019; Li X. et al., 2022).

##### 3.1.1.5 Metal chelating agents

These are defined as chemical compounds that bind tightly to metal ions. Presently metal chelating agents are being engaged as inhibitors against resistant bacteria. These agents can selectively disturb the essential metal metabolism of microorganisms by interfering with metal acquisition and bioavailability for crucial reactions. The chelation activity can inhibit the biological role of metal-dependent proteins like metalloproteases and transcription factors, disturbing the microbial cell homeostasis and culminating in the blockage of microbial nutrition, growth, and development, cellular differentiation, adhesion to biotic (e.g., extracellular matrix components, cell and/or tissue) and abiotic (e.g., plastic, silicone, and acrylic) structures as well as controlling the *in vivo* infection progression. Interestingly, chelating agents also potentiate the activity of classical antimicrobial compounds. Some of the examples of metal chelating agents which are active inhibitors of Metallo dependent *β*-lactamases (MBLs) like NDM, VIM, and IMP are 1,4,7-triazacyclononane-1,4,7-triaceticacid, 1,4,7,10-tetraazacyclod-odecane and 1,4,7,10-tetraacetic acid (La Piana et al., 2021; Somboro et al., 2019). As of now, the metal chelators 1,4,7-triazacyclononane-1,4,7-triacetic acid (NOTA), 1,4,7,10-tetraazacyclododecane (cyclen), and 1,4,7,10-tetraacetic acid (DOTA) are not used as drugs in clinical practice. Their potential therapeutic use requires extensive clinical trials to assess the safety, efficacy, pharmacokinetics, and pharmacodynamics of these chelators. The rise in resistance to combination agents, such as β-lactam and β-lactamase inhibitor combinations, is a growing concern in the management of bacterial infections. Although these combinations, like amoxicillin-clavulanic acid and piperacillin-tazobactam, have been effective in restoring the efficacy of β-lactam antibiotics against resistant bacteria, the emergence of new resistance mechanisms poses significant challenges, e.g., new β-lactamases, such as NDM-1 and KPC (*Klebsiella pneumoniae* carbapenemase), and mutations in existing enzymes can degrade both β-lactams and their inhibitors, reducing the effectiveness of these therapies (Morales-Durán et al., 2024).

##### 3.1.1.6 Inhibitors of modifying enzymes

Antibiotics such as aminoglycosides inhibit protein synthesis in bacteria by binding to the 70S ribosome (16S ribosomal RNA of 30S ribosome) of bacterial ribosomes leading to erroneous translation. Resistance to aminoglycoside antibiotics occurs by alterations in cell permeability, target modification, efflux, and mostly by enzymatic inactivation. The enzymes involved in the inactivation of aminoglycosides are known as Aminoglycoside-modifying enzymes (AMEs). AMEs are bacterial enzymes that confer resistance through the chemical modification of the aminoglycosides. They catalyze the modification of the 2-deoxystreptamine nucleus or the sugar moieties at different hydroxyl (-OH) or amine (-NH₂) groups via acetylation, phosphorylation, or adenylation (Ramirez and Tolmasky, 2010). The modifying enzymes of aminoglycosides are divided into three groups: (a) aminoglycoside acetyltransferases (AACs), which use acetyl CoA (donor substrate) to catalyze the acetylation of primary amine groups of aminoglycoside antibiotics, e.g., resistance against antibiotics paromomycin, lividomycin, gentamicin, sisomicin, fortomicin, netimicin, tobramycin, dibekacin; (b) aminoglycoside nucleotidyl transferases (ANTs) which use ATP (donor substrate) to transfer its adenosine monophosphate group to OH group of aminoglycoside molecules, e.g., resistance against antibiotics like dibekacin, tobramycin, amikacin, and isepamicin; and (c) aminoglycoside phosphotransferases (APHs) which catalyze the phosphorylation of OH group of aminoglycosides using ATP (Krause et al., 2016; Laws et al., 2019). Using bisubstrate compounds as inhibitors of aminoglycoside-modifying enzymes is emerging as a promising scheme to fight against aminoglycoside resistance. This therapy has proved fortunate in the case of gentamicin. Aminoglycosides are combined with CoA and various functional groups to make it an effective antibiotic. Aminoglycosides combined with CoA or its derivatives include chemical modifications to improve drug properties, such as cellular uptake and stability. Functional group modifications enhance aminoglycosides’ effectiveness against bacterial infections and resistance mechanisms. They are usually effective against AACs like AAC (3), and AAC (6′) (Laws et al., 2019; Magaña et al., 2023) Kanamycin conjugated with CoA is an effective against AAC (6′) in *E. faecium* (Zárate et al., 2018). Aminoglycoside-CoA conjugates with sulfone and sulfoxide functional groups are efficacious against AAC (6′) (Laws et al., 2019). Another potent inhibitor is Bovine peptide indolicidin which is effective against a broad range of aminoglycosides antibiotic resistance enzymes (APH and AAC) (Boehr et al., 2003; Magaña et al., 2023). APHs are structurally similar to protein kinases of eukaryotes and some of their inhibitors are also similar to compounds of the Isoquinoline sulfonamide group (Oruganty et al., 2016; Fong et al., 2011). Pyrazolopyrimidine compounds are also emerging as good inhibitors against APH (3′) enzymes found in most gram-negative bacteria (Liu et al., 2019; Stogios et al., 2013). While these strategies represent promising approaches, aminoglycosides still face challenges from AMEs. AME inhibitors are not yet in clinical use, primarily due to several significant challenges. Despite ongoing research, effective AME inhibitors have not been developed to the point of clinical application. The difficulties in creating these inhibitors stem from the complexity and variability of AMEs, which have diverse mechanisms and binding sites, making it hard to design broad-spectrum inhibitors. Additionally, many potential inhibitors face issues related to specificity, pharmacokinetics, and toxicity, which complicates their development and approval. The economic burden of drug development and the rigorous regulatory requirements further impede their introduction into the market. Although some AME inhibitors have shown promise in preclinical animal models, translating these results to humans remains challenging due to differences in drug metabolism and bacterial resistance mechanisms. The combination of these scientific, technical, and economic barriers has thus far prevented AME inhibitors from becoming a practical solution in clinical settings and ongoing research is necessary to develop more effective treatments (Böttger and Crich, 2020).

##### 3.1.1.7 Use of inhibitors of efflux pump

Efflux pumps are the transport proteins present in membranes of bacteria and almost every cell including eukaryotes. Both gram-positive and gram-negative bacteria engage efflux pumps to deploy resistance against antibiotics. The efflux pumps are categorized into five major families namely: ATP binding cassette (ABC), small multidrug resistance family (SMR), RND, multidrug and toxin extrusion family (MATE), and major facilitator superfamily (MFS). The efflux pump of gram-negative bacteria is structurally more complex than those present in gram-positive bacteria, due to the differences in their cell envelope structure. Gram-negative bacteria have a more intricate cell envelope with an inner and an outer membrane, necessitating efflux pumps that span both membranes, often forming tripartite systems. They comprise three proteins, namely, resistance nodulation division (RND) protein which acts as a transporter (activated by proton motive force) located in the inner membrane; outer membrane factor (OMF) immersed in the outer membrane and membrane fusion protein (MFP) located in the periplasm (Gaurav et al., 2023; Huang et al., 2022).

Efflux pumps expel a variety of substances including heavy metals, antiseptics, antibiotics, toxins, virulence factors, etc. The regulation of these efflux pumps is connected to many other regulatory mechanisms involved in virulence, e.g., quorum sensing, membrane permeability, and biofilm formation. These efflux pumps are considered a promising target for the establishment of various adjuncts that can be helpful in making bacteria sensitive to antibiotics again (Impey et al., 2020). Several properties should be satisfied by a compound to become a successful efflux pump inhibitor (EPI). The entity should be selective, non-toxic, cost-effective, and most importantly, non-antimicrobial itself. It should not attack any other efflux pumps of eukaryotes i.e.; they should inhibit selectively. The most popular efflux pump inhibitor is CCCP (Carbonyl cyanide-m-chlorophenylhydrazone) which disturbs the proton motive force (PMF) and also, the metabolism of cells. It has potentialized antibiotics like tetracycline and several carbapenems (Sharma A. et al., 2019; Sanchez-Carbonel et al., 2021; Rhee et al., 2016; Ardebili et al., 2014). IITR08027 is another popular artificial efflux pump inhibitor that has restored bacterial sensitivity towards ciprofloxacin and other fluoroquinolones. It acts by disrupting the proton gradient which leads to the inhibition of the AbeM-type MATE efflux pump (Bhattacharyya et al., 2017). Although CCCP is extensively used in *in-vitro* studies, challenges such as toxicity, and efficacy limit them from current clinical usage. Using EPI with antibiotics is a combination therapy that demands compatibility between EPI and antibiotics. For example, verapamil, a known calcium channel blocker, was used to treat hypertension and was discovered to inhibit efflux pumps. A retrospective study identified that when verapamil was combined with the macrolide antibiotic clarithromycin, it led to adverse effects such as kidney failure and hypotension due to the accumulation of verapamil to toxic levels. This drug combination is not used in clinical practice for infection treatment. The toxicity was observed in patients who were already on calcium channel blockers and subsequently prescribed clarithromycin, rather than from the use of this combination as a treatment regimen (Clifford et al., 2022; Sharma A. et al., 2019) Another challenge for EPIs is that they are quite specific for the restricted number of substrates. Moreover, the efflux pumps are not exclusively responsible for resistance in several bacteria, for example, in the case of *P. aeruginosa* and *A. baumannii*, efflux pumps along with mutation in gyrase coding genes are associated with the action of resistance (Abdi et al., 2020). Therefore, more efforts are required to accomplish these challenges and the effective use of these EPIs to combat antibiotic resistance with a high success rate. Some of the well-reported EPIs, their targets, and their effects are reported in Table 2. The compounds listed in the table, including natural and artificial efflux pump inhibitors such as carnosic acid, carnosol, geraniol, epicatechin gallates, and various synthetic derivatives, exhibit significant potential to enhance the efficacy of antibiotics by targeting bacterial efflux pumps. Despite their promise demonstrated in preclinical studies, these compounds have not yet transitioned to clinical practice. The journey from laboratory success to clinical application involves overcoming several challenges. Safety and toxicity must be thoroughly evaluated through preclinical studies and clinical trials to ensure these compounds do not cause adverse effects in humans. Additionally, optimizing pharmacokinetics and bioavailability is crucial for effective delivery and action in the human body. Regulatory approval processes also demand rigorous testing and validation to meet health authorities’ standards. The development of stable and effective formulations, along with securing funding and forming strategic collaborations, is essential for advancing these compounds. Addressing these challenges is key to moving from promising research findings to practical clinical applications, potentially offering new solutions in the fight against antibiotic resistance.

**Table 2:** Table showing different EPIs and their target efflux pump.

Origin	Class	Example	Efflux pumps they target	References
Natural	Phenolic diterpenes	Carnosic acid (They provide efficacy to tetracycline and erythromycin.)	ABC Transporter MsrA and TetK efflux pumps	Sharma A. et al. (2019)
		Carnosol (Derivative of carnosic acid)		
	Monoterpenoid alcohol	Geraniol (Reduce the MIC of Chloramphenicol.)	Tripartite efflux pump (AcrAB-To1C)	Sharma A. et al. (2019)
	Polyphenols	Epicatechin gallates (Decrease the MIC of Oxacillin.)	NorA efflux pumps.	Sousa et al. (2019), Sharma A. et al. (2019)
		Epigallocatechin (Potentiate Tetracycline, erythromycin, and ciprofloxacin.)	Inhibits NorA efflux pumps (with less potency) Also inhibits TetK in gram-positive Staphylococci and gram-negative Campylobacter spp.	Magaña et al. (2023), Nguyen et al. (2023)
	Flavones and Isoflavones	Baicalein (Refined sensitivity of MRSA towards Ciprofloxacin and β-lactams. Also, improved efficacy of tetracycline.)	Inhibits TetK-overexpressing Staphylococci and NorA efflux pumps.	Waditzer and Bucar (2021)
	Alkaloids	Piperine (Re-sensitized bacteria of *S. aureus* strains towards Ciprofloxacin.)	Inhibits the functioning of ABC transporters. Inhibit NorA efflux pump.	Wink et al. (2012), Pule et al. (2015)
		Reserpine (Potentiate tetracycline and norfloxacin.)	Mostly effective against efflux pumps of RND and MFS families. Inhibits NorA and Bmr efflux pumps	Dewanjee et al. (2017), Shriram et al. (2018)
	Homoisoflavonoids	Bonducellin (Shows synergic behavior with ethidium bromide.)	Inhibits multidrug resistance efflux pumps.	Castelli and López (2017), Roy et al. (2013)
Artificial	Quinolone derivatives	Pyridoquinolones derivatives (Potentialize norfloxacin, tetracycline.)	Inhibition of AcrAB-To1C efflux pumps. Also, inhibits the RND pump.	Laws et al. (2019)
	Peptidomimetics	Phenylalanine-arginine-β-naphthylamide (PAβN) (Decreased the MIC of Levofloxacin. Restored the activity of fluoroquinolones, macrolides and chloramphenicol.)	Inhibits RND efflux pumps. MexAB-OprM, MexCD-OprJ and MexEF-OprN pumps.	Laws et al. (2019)
	Arylpiperazine	1-(1-Naphthylmethyl)-piperazine (NMP) Potentiate Levofloxacin.	Inhibition of RND pumps. Inhibits AcrAB and AcrEF efflux pumps.	Laws et al. (2019)

### 3.2 Inhibition of quorum sensing

The process of cell-to-cell communication present in bacteria to synchronize the pathogenic behaviors and to also, evoke biological response against stimulus by expression of multiple genes is called Quorum Sensing. Quorum-sensing signals are triggered by extracellular chemical signals produced by the biofilm itself. Biofilm formation is one of the well-known causes of antibiotic resistance. It acts as an effective barrier to drugs or antibiotics by decreasing their permeability. The changes in the external environment like changes in pH, temperature, and chemical concentration lead to the regulation of its physiological functions and resistance to antibiotics. Near about 65% of infectious bacteria grow as biofilms and become 10 to 1,000 times more resistant to antibiotics at their pathogenic stage (Sharma D. et al., 2019). A prime example of this is the case of *P. aeruginosa*. For the development of pathogenicity in *P. aeruginosa* biofilm formation and quorum sensing are vital and are of great importance (Rather et al., 2021). Some vital signals are acyl-homoserine lactones (AHLs), autoinducing peptides (AIPs), and autoinducer-2 (AI-2). Gram-negative bacteria use AHLs whereas gram-positive bacteria use peptides as their signal molecules (Verbeke et al., 2017).

Autoinducers (AIs) are signaling molecules used in quorum sensing (QS) systems to regulate gene expression in response to cell density. These molecules facilitate communication between bacteria, allowing them to coordinate behavior on a community-wide scale. Different autoinducers are used by various bacterial species, and they play crucial roles in processes such as biofilm formation, virulence, and antibiotic resistance. Some examples of AIs are: Acyl-Homoserine Lactones, (AHLs)(AI-1). They are generally N-acyl homoserine lactones (AHLs), which have a lactone ring attached to an acyl side chain, e.g., N-3-oxo-hexanoyl-L-homoserine lactone (3-oxo-C6-HSL) in *P. aeruginosa*, N-hexanoyl-L-homoserine lactone (C6-HSL) in *Vibrio fischeri.* AI-1 molecules bind to specific receptor proteins in the same bacterial cell or in neighboring cells, leading to changes in gene expression. They are typically used in gram-negative bacteria. Autoinducer-2 (AI-2) is a more complex signaling molecule, often a furanosyl borate diester derivative. AI-2 is produced by a wide range of bacterial species and is involved in interspecies communication. It acts through a receptor system that is often involved in regulating diverse behaviors such as biofilm formation and virulence, e.g., (S)-4,5-Dihydroxy-2,3-pentanedione (DPD) in *Vibrio harveyi*, (2S,3S)-2,3-dihydroxybutane-1,4-dione (DHB) in other species. Autoinducer-3 (AI-3) is structurally less defined compared to AI-1 and AI-2 but is known to be involved in bacterial communication. AI-3 interacts with the LuxS system and is involved in regulating virulence and biofilm formation in gram-negative bacteria, e.g., AI-3 in *E. coli.* A classic quorum sensing regulatory circuit system can be observed in *Vibrio* species like *V. fischeri.* The primary QS system involves the production of N-acyl homoserine lactones (AHLs). In *V. fischeri,* the LuxI protein synthesizes the AHL signal, and LuxR is the receptor that binds to the AHL. When the concentration of AHL reaches a threshold, LuxR binds to the AHL, forming a LuxR-AHL complex that activates the transcription of genes involved in bioluminescence. This system controls bioluminescence in *V. fischeri*, which is used for symbiotic relationships with marine animals. Similarly, *V. harveyi* uses multiple autoinducers, including AI-1, AI-2, and AI-3. It has a more complex regulatory circuit system involving the LuxN receptor for AI-1 and the LuxS system for AI-2. The LuxN and LuxS systems interact to regulate gene expression based on the concentration of autoinducers. This system regulates bioluminescence and virulence factors, adjusting the bacterial behavior based on cell density and environmental conditions (Frezza et al., 2007; Coquant et al., 2020; Gill et al., 2015; Wang et al., 2019; Mayer et al., 2023; Papenfort and Bassler, 2016).

QS plays a significant role in biofilm formation by regulating genes involved in adhesion, biofilm matrix production, and bacterial aggregation. High cell density triggers the QS systems, leading to the expression of genes that promote biofilm development. The biofilm structure protects bacteria from environmental stresses and antimicrobial agents, e.g., in *P. aeruginosa*, QS regulates the production of exopolysaccharides that are crucial for biofilm matrix formation. The LasR-LasI and RhlR-RhlI systems control these processes through the production of AHLs. In multispecies biofilms, different bacterial species may use distinct QS systems that can interact with each other, e.g., *V. cholerae* and *P. aeruginosa* can form mixed-species biofilms where their QS systems influence each other’s behaviors. The interaction of various autoinducers in a biofilm can lead to complex regulatory networks that affect biofilm stability, antibiotic resistance, and nutrient availability (Hoang et al., 2022).

Autoinducers are critical signaling molecules in quorum sensing systems that regulate bacterial behavior in response to population density. In biofilms, QS systems orchestrate complex interactions among bacteria, affecting biofilm formation, maintenance, and resistance. Understanding these systems offers insights into bacterial communication and potential strategies for disrupting biofilm-associated infections (Kumar et al., 2022).

Quorum sensing inhibitors (QSIs), also known as quorum quenchers, inhibit the process of quorum sensing which helps in reducing the risk of antibiotic resistance (Zhong and He, 2021). The process of interfering with the quorum sensing system of bacteria by obstructing the signaling between them is called quorum quenching. Quorum quenching can be categorized into three main classes: (1) inhibition of production of a signaling molecule, (2) degradation of a signaling molecule, and (3) inhibition of conduction of a signaling molecule or binding to the receptor (Zhou et al., 2020). Some of the examples of quorum-sensing inhibitors and their biological effect are shown in Table 3.

**Table 3:** Quorum sensing inhibitors along with their biological effect.

Function of quorum sensing inhibitor		Example of inhibitors	Biological effect	References
Obstructing the production of signaling molecules	AHL production blocking	Butyryl-S adenosylmethionine, L/D-S-adenosylhomocysteine	Inhibits production of AI-1 signal molecules.	Zhao et al. (2020)
	Inhibition of the LuxS enzyme activity	TNRHNPHHLHHV (peptide)	Inhibits the AI-2 signal molecules production.	Wang et al. (2019)
	Inhibitor off 5′-Methylthioadenosine Phosphorylase (MTAP)	MT-DADMe-ImmA	Blocks the production of AI-2 signal molecules.	Zhao et al. (2020)
	Inhibition of enzyme activity responsible for signal molecule synthesis	FabI derivatives (used for fatty acids biosynthesis)	Inhibit the production of signal molecules	Kalia et al. (2019)
Destruction of Signalling molecules	AHL signalling molecule degradation	aiiA gene in *Bacillus* sp. *240B1*, AHL lactonase (AidB), BbMoml (a recombinant strain)	Enzymatic degradation of AHL molecule	Zhao et al. (2020)
	Autoinducer-2 signal molecule degradation	Imidazole	Inhibition of AI-2 molecule	Yu et al. (2018)
	AI-2 signal molecule phosphorylation and degradation	Addition of ATP and LsrK	Reduced QS	Shi et al. (2023)
Inhibition of binding of a signalling molecule with the receptor	Competitors of AI-2 signal molecules	D-Galactose	Inhibits AI-2 system	Ryu et al. (2016)
	Decrease the concentration of QS Signal	Flavonoids	Inhibits quorum sensing	Paczkowski et al. (2017)
	Prevention of homodimers formation	Small peptide 5,906	Inhibition of LuxS activity	Sun and Zhang (2016)
	Inhibits the signalling	Alkyl-Quinoxalin-2(1H)-one derivatives	Inhibition of formation of biofilm	Blöcher et al. (2018)
	Disruption of Wnt pathway	Derivatives of 2H-pyran-3(6H)-one	Inhibit Wnt/β-Catenin signalling	Zhao et al. (2020)

Quorum-quenching enzymes inactivate the signaling whereas QSIs are the chemical entities that disturb the quorum-sensing pathway (Zhao et al., 2020; Verbeke et al., 2017). A perfect QSI should have several characteristics like they should be highly specified for regulators involved in quorum sensing. The compound should be of low molecular mass and non-toxic to host cells. They should not obstruct the metabolic activities of bacterial cells like DNA metabolism, protein synthesis, etc. and most importantly, they should be chemically stable (Asfour, 2018). A well-known example of QSI is Furanones which imitates the quorum sensing signaling molecules of AI-2 and AHL-based QS systems structurally and therefore, inhibits it. An example of effective furanones is seen in the bacteria *Vibrio harveyi,* in which halogenated furanones prevent the binding of LuxR with DNA which further prevents the transcription (Paczkowski et al., 2017). Furanone-4 has potentialized tobramycin against *P. aeruginosa* and brominated furanones have been observed as highly efficacious against fungi like *Candida albicans,* both gram-positive and gram-negative bacteria such as *S. aureus*, *S. enterica* and *S. maltophilia* (Gómez et al., 2022). Small molecules like triclosan act as an inhibitor of the enzyme, enoyl-ACP reductase which is an important intermediate component used in the synthesis of AHLs (Chakraborty et al., 2023). Analogs of HSL (signal molecule of QS) are used as competitors to bind with receptors and block quorum sensing (Zhou et al., 2020). Quorum quenching enzymes can also inhibit biofilm formation and revert to their sensitivity towards antibiotics, for example, AHL lactonase, AHL acylase, etc. A novel antimicrobial peptide, Peptide 1018, can be combined with antibiotics like tobramycin, ceftazidime, imipenem, and ciprofloxacin for inhibition of biofilm formation (Gill et al., 2015; Alzahrani et al., 2022). Several AI-2 analogs can be useful in blocking the binding of QS signalling molecules with their receptor. Analogs of DPD and boronic acid are used as competitors of autoinducer-2 (Papenfort and Bassler, 2016).

Although quorum sensing inhibition is one of the potential strategies to combat antibiotic resistance it has its limitations and challenges like late introduction of drug resistance in the field of quorum sensing, incomplete studies, and lack of knowledge regarding the regulatory mechanism of microbial resistance. Advantages of QSIs include the potential to reduce the severity of infections by lowering the production of virulence factors, disrupting biofilms that are difficult for traditional antibiotics to penetrate, and enhancing the effectiveness of existing antibiotics through synergistic effects. Additionally, QSIs have the potential for broad-spectrum activity against various bacterial species. However, there are several disadvantages associated with QSIs. They are still largely in preclinical and early clinical stages, meaning there is limited data on their long-term safety and efficacy. There is also a risk of bacteria developing resistance to QSIs, similar to traditional antibiotics. Formulation and delivery challenges, along with high development costs, may affect their practical use and accessibility. Moreover, QSIs could unintentionally impact beneficial bacteria in the human microbiome, leading to potential dysbiosis. Finally, the complexity of targeting diverse quorum-sensing systems across different bacteria adds to the development challenges. Moreover, studies on quorum sensing, its role in pathogenicity and virulence, and how it affects the development of antibiotic resistance in microorganisms are limited to only a few bacterial pathogens such as *P. aeruginosa, Vibrio cholerae, Brucella abortus, Acinetobacter,* etc. and are poorly understood. Even with the persisting limitations and challenges, studies do show that quorum sensing affects the expression of pathogenicity, along with a key role in biofilm formation and drug efflux pump overexpression. Thus, making quorum sensing a direct avenue in controlling and preventing infections. The use of QSI simultaneously with antibiotics will help not only reduce resistance in microorganisms but also help increase the bactericidal efficacy of antibiotics (Zhao et al., 2020; Castillo-Juárez et al., 2015).

### 3.3 Effective absorption and action of antibiotics

The dwindling amount of effective antibiotics available for treatment and the absence of new antibiotics is a hindrance to the effective treatment of bacterial infection (Dutescu and Hillier, 2021). Since the development of any new and effective antibiotic is a time-consuming process, it’s the need of the hour to develop methods for effective absorption and action of already existing antibiotics (Miethke et al., 2021). Some of those strategies are discussed below.

#### 3.3.1 Use of membrane permeabilizers for effective penetration and action of antibiotics

The need for new entities that could enhance the movement of antibiotics inside the cell is inevitable in today’s scenario of increasing antibiotic resistance. For this, several chemosensitizers have been introduced to combine with antibiotics and increase their efficacy to combat antibiotic resistance. Chemosensitizers can alter the permeability of a bacterial cell membrane or prevent the bacteria from removing antibiotics via efflux pumps. Therefore, chemosensitizers may be able to combat antibiotic resistance by helping antibiotics cross bacterial membranes and accumulate inside bacterial cells (Lôme et al., 2018). The cell wall of gram-negative bacteria is distinct from that of gram-positive bacteria as it has an outer hydrophobic bilayer membrane. It consists of membrane proteins (pore-forming proteins), phospholipids, and lipopolysaccharides. The cross-linking of the LPS layer by divalent cations (Mg^2+^ and Ca^2+^) is responsible for its stability. This outer layer makes gram-negative bacteria less susceptible to many antibiotics by acting as a barrier against them. The small molecules of antibiotics follow two mechanisms to cross through the cell wall. Hydrophobic antibiotics like macrolides and rifampicin penetrate the bilayer using the mechanism of passive transport whereas aminoglycosides and chloramphenicol enter by diffusion. Antibiotics like fluoroquinolones, *β*-lactams, and chloramphenicol are hydrophilic and undergo the mechanism of active transport with the help of porins to cross the lipid bilayer (Prajapati et al., 2021). Thus, the bacterial cell walls can be efficiently exploited as a target for the therapy of antibiotic resistance. Various membrane permeabilizers can be used in combination with antibiotics to increase the chances of entry for antibiotics. These permeabilizers are cationic chelating agents (chelate Mg^2+^ and Ca^2+^) that interact with anionic lipopolysaccharides and destabilize the cell wall to allow the entry of antibiotics through it. Though cationic chelators, while effective in various applications, face significant challenges such as toxicity, incompatibility with biological systems, lack of selectivity, poor absorption, and low stability when considered for use as drugs in humans. Polymyxins, liposomal drug preparations, and some cationic peptides are the best membrane permeabilizers used nowadays to increase the influx of antibiotics. Polymyxins are the most commonly used membrane permeabilizers for treating resistant bacteria. They are the lipopeptide compounds with a cyclic peptide and fatty acid chain. They are penta cationic and link with the outer membrane electrostatically to remove divalent cations (Mg^2+^ and Ca^2+^) from their interaction sites to destroy the coherence of the membrane which results in enhanced influx of antibiotics (Ayoub Moubareck, 2020; Ledger et al., 2022). Polymyxin B and Polymyxin E (colistin) are also well-known antibiotic adjuvants that disturb the integrity of the outer membrane of gram-negative bacteria (Ayoub Moubareck, 2020). Colistin is used in combination with many other antibiotics like rifampicin as an additive against *A. baumannii* to combat its antibiotic resistance (Armengol et al., 2020). PMBN (polymyxin B nonapeptide) is another prominent membrane permeabilizer useful in reawakening the sensitivity to hydrophobic antibiotics in bacteria (Trimble et al., 2016). Besides this, polymyxin decapeptides, colistin nonapeptides, polymyxin B octapeptides, and polymyxin B heptapeptides are all good outer membrane permeabilizers (Ayoub Moubareck, 2020). Polymyxin B analog-SPR741 shows successful synergy with clarithromycin and rifampicin and has also completed the clinical phase I trials. They are underway for further clinical investigation (Corbett et al., 2017). Another second-generation derivative of polymyxin is NAB741, which made *E. coli* sensitive to azithromycin, mupirocin, and vancomycin again and also, showed synergy with rifampicin and clarithromycin in *E. coli*, *A. baumannii* and *K. pneumoniae* (Vaara, 2019). Another example of membrane permeabilizers is Octapeptins. It is a rediscovered class of lipopeptides and is useful in permeabilizing the outer membrane of bacteria and making them sensitive to antibiotics. They are structurally analogous to polymyxins (Leu residue present at C-terminal instead of polymyxin Thr residue) such as Octapectin C4 (an amphiphilic antibiotic), which shows the same fluorescence level as polymyxin B and colistin. They bind with the outer membrane and depolarize to enter the cell. Octapeptins were first found in *Bacillus circulans* and *Paenibacillus tianmuensis* almost 40 years ago. These lipopeptides act against polymyxin-resistant XDR gram-negative bacteria. They embed more strongly into OM and remain active even when modifications to lipid A inhibit polymyxin binding. They do not show any cross-reactivity to polymyxins and hence, can be used efficiently in therapy against antibiotic resistance (Blaskovich et al., 2018). The clinical use of polymyxins has been limited by their toxicity profile. Common adverse effects include nephrotoxicity and neurotoxicity. These side effects are particularly concerning given that polymyxins are often used in critically ill patients who may already have compromised renal function. The risk of toxicity has led to a cautious approach to their use, often reserving them for severe infections where alternative treatments have failed. To mitigate the risk of toxicity, several strategies such as dose optimization, combination therapy, and the development of new formulations have been employed to reduce systemic exposure and associated toxicities. Despite their limitations, polymyxins remain a valuable option for treating multidrug-resistant infections. Balancing their use with careful monitoring and innovative approaches to minimize toxicity will be crucial in maintaining their utility in the clinical arsenal. Ongoing research focuses on optimizing their use, improving safety profiles, and developing alternative agents with similar efficacy but fewer side effects. Antimicrobial peptides (AMPs) represent a diverse group of molecules that form part of the innate immune system across a wide range of organisms. These peptides are typically small, cationic, and amphipathic, enabling them to interact with microbial membranes and exert a broad spectrum of antimicrobial activity. The ability of AMPs to target multiple bacterial structures and processes makes them less prone to resistance, thus making them attractive candidates for combating antibiotic-resistant infections. AMPs can be classified into several types based on their structure and function: (a) *α*-Helical peptides that disrupt bacterial membranes by forming pores or by destabilizing the lipid bilayer, leading to cell lysis, e.g., magainins and melittin. (b) *β*-Sheet peptides which form pore-like structures in microbial membranes causing loss of membrane integrity, e.g., defensins. (c) Extended peptides are those peptides that are rich in specific amino acids (e.g., tryptophan) and can insert themselves into the membrane, causing disruption or translocating into the cytoplasm to target intracellular components, e.g., indolicidin. And (d) Looped Peptides are those peptides that contain disulfide bridges, thus contributing to the stabilization of their structure and enhancing their interaction with bacterial membranes, e.g., bactenecin. The broad-spectrum activity and ability to target bacteria in ways distinct from traditional antibiotics, AMPs hold significant potential for treating infections caused by multidrug-resistant (MDR) and extensively drug-resistant (XDR) bacteria. Specific AMPs have been explored for their abilities such as disruption of biofilm, synergistic effects with traditional antibiotics, and specificity toward targeted pathogens. Despite their potential, several challenges such as stability, bioavailability, toxicity, immunogenicity, and cost of production need to be addressed before AMPs can be widely used in clinical practice (Gao et al., 2021; Drayton et al., 2021; Lei et al., 2019; Mishra et al., 2018).

Another approach used against antibiotic resistance through membrane permeabilization is the use of peptidomimetics and ethylene diamine tetra acetic acid. Peptidomimetics are compounds resistant to enzymatic inactivation or degradation and imitate the antibacterial peptide’s mode of action of permeabilization of the outer membrane. C_12_K-7_α8_ is a peptidomimetic compound with less hemolytic activity and potentiates antibiotics erythromycin, clarithromycin, and tetracycline (Molchanova et al., 2017). LL-37, a cathelicidin peptide found in humans is an antimicrobial and increases the efficacy of macrolide antibiotic (azithromycin) (Lin et al., 2015). EDTA, a chelating agent liberates a large amount of lipopolysaccharides from the outer membrane by chelating Mg^2+^ and Ca^2+^, destroying its integrity (Paracini et al., 2022). Another cationic polymer, polyethyleneimine (PEI) increases the sensitivity of some strains of *Pseudomonas* toward antibiotics like erythromycin, novobiocin, and fucidin (Liu et al., 2021; Pandey et al., 2023). Carrageenin is another class of adjuvants that acts as cationic steroidal antibiotics (CSA), capable of forming complexes with phospholipids of the outer membrane. This class consists of CSA-8 and CSA-13, which have some properties similar to antimicrobial peptides, due to which; they make bacteria more sensitive to hydrophobic antibiotics (Svenson et al., 2022). Many peptidomimetics have shown promise in preclinical studies and early-phase clinical trials.

Polyethyleneimine (PEI) and carrageenin-based adjuvants (CSA-8 and CSA-13) are promising tools in the fight against antibiotic resistance, particularly by enhancing the efficacy of antibiotics against bacteria like *P. aeruginosa*. PEI increases bacterial sensitivity to antibiotics such as erythromycin and novobiocin, while carrageenin derivatives can disrupt bacterial membranes, making them more susceptible to hydrophobic antibiotics. However, both compounds are still in the preclinical phase of development, with no large-scale clinical trials currently underway. The practicality of using these adjuvants lies in their ability to revitalize existing antibiotics, offering a broad-spectrum approach that could combat resistant strains. Despite their potential, significant challenges remain, including concerns about toxicity, formulation stability, and ensuring *in vivo* efficacy. The complexity of translating these findings into clinical practice is further compounded by the need for extensive safety testing and regulatory approval. Therefore, while PEI and carrageenin-based adjuvants hold promise, further research is essential to overcome these challenges and fully realize their potential in clinical settings (Afrasiabi and Partoazar, 2024).

#### 3.3.2 Use of nanoparticles as delivery agents of antibiotics

In the case of many antibiotics, resistance develops due to the inability of antibiotics to cross the membrane rendering them inefficient. To overcome this problem nanoparticle-loaded antibiotics are being administered. The use of nanoparticles as a medium to cross the membrane of bacteria can improve the efficacy of antibiotics (Makabenta et al., 2021). Factors like particle size, surface charge, and solubility of nanosystems play key roles in this approach (Ozdal and Gurkok, 2022). Metallic nanoparticles such as Ag-NP are the prominent carriers of antibiotics. One of the most significant uses of nanoparticles is that multiple drugs can be loaded on them (Chandrakala et al., 2022). Besides acting as transporters, NPs themselves inhibit the formation of biofilm, activate immune responses, and damage DNA and proteins (efflux pump proteins) of bacteria with the help of ROS (reactive oxygen species) response. For example, AgNPs disrupted biofilms formed by *P. aeruginosa* and *S. aureus* by generating ROS, which caused significant damage to bacterial DNA and cell membranes, ZnO NPs effectively inhibited biofilm formation by multidrug-resistant *E. coli*, (TiO_2_) NPs, which not only produce ROS but also activate immune responses, further enhancing their antibacterial effects. Copper oxide (CuO) and iron oxide (Fe_3_O_4_) NPs similarly contribute to the disruption of biofilms by affecting bacterial adhesion and efflux pump function (Makabenta et al., 2021; Gupta et al., 2019; Hetta et al., 2023; Afrasiabi and Partoazar, 2024). Based on their origin nanoparticles are classified into two classes, namely, organic and inorganic nanosystems. Organic nanosystems like polymeric micelles, liposomes, solid lipid nanoparticles, etc. are derived from organic compounds whereas inorganic nanosystems like metallic nanoparticles are derived from inorganic oxides. The use of NP in combating antibiotic-resistant bacteria presents promising advancements in antimicrobial therapies but their clinical translation presents both opportunities and challenges. Organic nanosystems, such as conventional liposomes composed of dipalmitoyl phosphatidylcholine (DPPC), have demonstrated significant enhancement in antibiotic delivery, particularly against infections like *P. aeruginosa*. Nanoparticles show good consistency with surfactants as they stabilize the surface of nanoparticles (Cortés et al., 2021). Many resistant bacteria became sensitized after getting treated with drugs exposed to surfactants (Ceresa et al., 2021). SLNs (solid lipid nanoparticles) are the most significant nanoparticles of lipids. Also, amphiphilic polymers have a success rate in combating the resistance. Liposomes are also good ‘delivery boxes.’ It has been observed during *in vivo* studies that ofloxacin-loaded liposomes are effective against *P. aeruginosa,* and clarithromycin-loaded liposomes are effective against *H. pylori. In vitro* studies of rifabutin-loaded liposomes found increased effectiveness against *M. tuberculosis,* azithromycin-loaded liposomes are effective against *Burkholderia cepacia* complex, and clarithromycin-loaded liposomes are active against *Mycobacterium avium* complex (MAC) (Solleti, 2016; Rodenak-Kladniew et al., 2019; Costa et al., 2016; Lotfipour et al., 2016) (Eleraky et al., 2020). These liposomes improve drug stability, reduce systemic toxicity, and can overcome bacterial defenses by facilitating targeted delivery to the infection site. However, they are prone to stability issues in the presence of biological fluids, which can lead to premature drug release, reducing their therapeutic efficacy. Moreover, the scalability of liposome production with consistent quality remains a significant technical challenge. Cationic liposomes, such as those made from dimethyldioctadecylammonium bromide (DODAB), have shown efficacy against Gram-positive bacteria like MRSA by interacting with negatively charged bacterial membranes. However, their strong positive charge can induce cytotoxicity and provoke inflammatory responses in host tissues, raising safety concerns that require careful formulation optimization. Surface-modified liposomes, which are engineered by incorporating targeting ligands like wheat germ agglutinin, offer enhanced specificity towards bacterial pathogens, reducing off-target effects and potentially lowering the required therapeutic dose. Nonetheless, the complexity of designing these systems increases the cost and difficulty of production. Additionally, the potential immunogenicity of surface modifications necessitates further investigation to ensure biocompatibility.

Inorganic nanosystems, particularly metal-based nanoparticles like silver nanoparticles (AgNPs), have garnered attention due to their broad-spectrum antimicrobial activity. AgNPs, especially when combined with antibiotics like vancomycin, have proven effective against various MDR bacteria, including MRSA, VRSA, and ofloxacin-resistant *P. aeruginosa*. The bactericidal activity of AgNPs is primarily attributed to their ability to disrupt bacterial cell walls, generate reactive oxygen species (ROS), and interfere with bacterial DNA. However, the cytotoxic effects of AgNPs on human cells and concerns about environmental toxicity and the potential for bacteria to develop resistance to silver limit their clinical application. Zinc oxide nanoparticles (ZnONPs) exhibit antimicrobial activity through mechanisms that include cell wall disruption and ROS generation, making them effective against strains like ampicillin-and carbenicillin-resistant *Klebsiella pneumoniae* and MDR *Escherichia coli*. Despite their efficacy, the oxidative stress induced by ROS can also damage host tissues, necessitating a careful balance between antimicrobial activity and cytotoxicity. Gold nanoparticles (AuNPs) offer a unique advantage due to their inherent biocompatibility and ease of functionalization. They can penetrate bacterial biofilms and disrupt cell membranes, and they have shown effectiveness against vancomycin-resistant *S. aureus* and carbapenem-resistant *A. baumannii*. However, the high cost of gold and the technical challenges of precisely controlling the size, shape, and surface properties of AuNPs limit their widespread clinical use. Additionally, while gold is generally considered biocompatible, the long-term effects of AuNPs in the human body are not fully understood and require further research (Slavin et al., 2017; Mutalik et al., 2023; Tiwari et al., 2018; Huang et al., 2024).

The transition of nanoparticle-based therapies from the laboratory to clinical practice faces several significant hurdles. These include the challenges of large-scale production, achieving consistent quality control, navigating the regulatory landscape, and ensuring that these nanosystems can be safely and effectively delivered to the target site within the human body. While there is significant progress in preclinical studies, more *in vivo* studies and clinical trials are needed to evaluate the safety, efficacy, and long-term impact on patients and the environment. Finally, the potential for bacteria to develop resistance to nanoparticle-based treatments is a concern that warrants continuous monitoring and research to develop strategies to mitigate this risk. Some common examples of NP systems are given in Table 4.

**Table 4:** Different nanosystems and their target organisms.

Nanosystem		Example	Result	References
Organic	Conventional liposomes	Dipalmitoyl phosphatidylcholine (DPPC)	Enhanced activity against *P. aeruginosa* infection/biofilm	Castoldi et al. (2017)
		pH-sensitive lipids (PSLs): Phosphatidylcholine (PCS 100): Cholesterol (1:3:1 w/w/w)	Active against MRSA	Jadhav et al. (2018)
		Hydrogenated soy phosphatidylcholine and cholesterol (7:3 w/w)	Enhanced activity against *P. aeruginosa*	Khatib et al. (2019)
	Cationic liposomes	Dimethyldioctadecylammonium bromide (DODAB)	Active against MRSA	Rukavina et al. (2018)
	Fusogenic liposomes	Dope/Dppc/CHe MS (4:2:4 molar ratios)	Active against *S. epidermidis (A. baumannii)*	Nicolosi et al. (2015)
	Surface-modified liposomes	Phospholipid, cholesterol, tween 80, vitamin E (6:1:1.8:0.12 mass ratios)	Active against *E. coli S. aureus*	Zhang et al. (2019)
		Wheat germ agglutinin	*Active against Aggregatibacter actinomycetemcomitans*	Wijetunge et al. (2020)

Nanosystem		Example of targeted bacteria	Result	References
Inorganic	Silver nanoparticles (AgNP)	MRSA, VRSA, Ampicillin resistant *E. coli, S. aureus,* Ofloxacin resistant *P. aeruginosa* etc.	Cause bacterial cell death when combined with vancomycin	Saeb et al. (2014), Baptista et al. (2018)
	Gold nanoparticles (AuNP)	*Vancomycin-resistant S. aureus, Enterococcus faecalis*; Ampicillin resistant *S. aureus, P. aeruginosa, E. coli*; Carbapenems resistant *A. baumannii* etc.	*Disruption of cell wall (penetration through biofilm (DNA damage))*	Baptista et al. (2018)
	Zinc oxide nanoparticle (ZnONP)	Ampicillin-Carbenicillin resistant *K. pneumoniae*; MDR *E. coli* etc.	Disruption of cell wall and generation of ROS	Yeom et al. (2016), Cha et al. (2015)
	Silicon nanoparticle (SiNP)	MRSA	Disruption of cell wall	Zhao et al. (2017)
	Copper oxide nanoparticles (CuONP)	MDR *E. coli, S. aureus*	Generation of ROS	Baptista et al. (2018)
	Graphene oxide nanoparticle	MRSA, MDR *E. coli*, *S. aureus*	Multiple toxic mechanism	Baptista et al. (2018)

### 3.4 Plasmid curing of resistant bacterial strains

**Figure 3:**
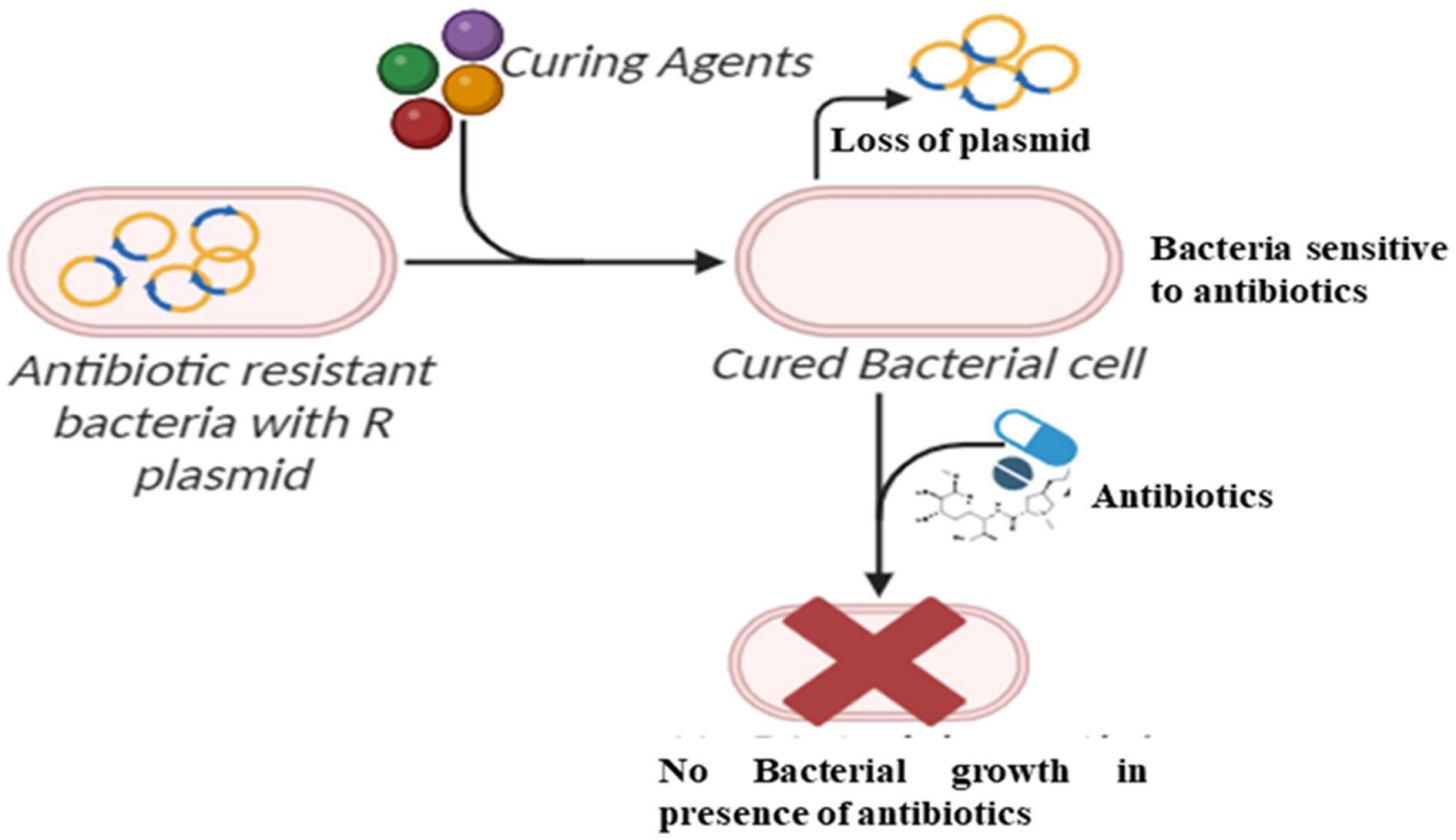
Plasmid curing to resensitize the resistant bacteria.

Antibiotic resistance genes are often located on plasmids and can disseminate in bacteria. These genes are the crucial elements responsible for the rise in the global spread of multidrug resistance (Coleman and Smith, 2014). Plasmids show a high rate of flexibility as they often undergo insertions, deletions, and repositioning of DNA elements, and this results in modifications in antibiotic-resistant genes (ARG), e.g., ESBLs (CTX-M), carbapenemases (NDM and OXA-58), etc. (Rafferty and Quinn, 2018). Plasmid curing and anti-plasmid techniques are useful in sensitizing the bacteria to antibiotics (Figure 3). The process of obviating the plasmid-encoded functions such as antibiotic resistance, virulence, degradation of aromatic compounds, etc. in bacteria is called Plasmid curing (Patwardhan et al., 2018). This approach has been explored for several decades, with studies focusing on various compounds including detergents (e.g., sodium dodecyl sulfate, bile), triclosan, ethidium bromide (EtBr), antibiotics (e.g., ciprofloxacin, ofloxacin, norfloxacin, rifampicin), phage therapies, and plant-derived compounds like 1′-acetoxychavicol acetate and plumbagin. More recently, the CRISPR/Cas system, an adaptive immunity mechanism in bacteria for targeting and destroying foreign DNA sequences, has also been utilized for plasmid curing. Regarding the effectiveness and application of plasmid curing, studies have reported the successful removal of plasmids carrying resistance genes in various bacterial species. For example, *in vitro* studies have demonstrated the curing of plasmids pSLT and pESI in *Salmonella enterica*, haemolysin plasmids in *E coli*, penicillinase plasmids in *S. aureus* and *E. coli*, and vancomycin resistance plasmids in *P. aeruginosa* (Buckner et al., 2018). While these *in vitro* results are promising, translating plasmid curing into clinical practice faces several challenges.

The practicality of plasmid curing in a clinical setting depends on factors such as the ability to target specific plasmids without affecting essential bacterial functions, the potential for developing resistance to the curing agents, and the effectiveness of these agents in diverse and complex environments like the human body. Additionally, the delivery of plasmid-curing agents *in vivo* remains a significant hurdle, as does ensuring the safety and selectivity of these treatments. Overcoming these challenges will require further research into the optimization of plasmid-curing compounds, improving delivery mechanisms, and understanding the long-term impacts on microbial communities.

### 3.5 Genomic studies of antibiotic resistance determinants\factors and antibiotic resistant microorganisms

The study of genomics is crucial in monitoring and controlling antibiotic resistance. They provide information about the existing resistance mechanisms, development, and microevolution of resistant strains and new resistance mechanisms. Genomic studies of different ecological ecosystems can help in the discovery of novel antibiotic synthetic genes from unrevealed bacteria present in those ecosystems, e.g., gene clusters for the production of antibacterial compounds, turbomycin A and B (glycopeptide) were discovered by cloning of environmental DNA libraries (Santana-Pereira et al., 2020). Functional and structural genomic studies contribute efficiently to understanding and identifying new targets for designing new and potent antibiotics. In context of genetics, resistance emerges either due to the gene expression of resistance contributing factors or by mutations. Tracking down the epidemiology of resistant bacteria and genes responsible for resistance is essential to assess its threat in terms of pathogenesis. Moreover, the knowledge of genomics can help to establish molecular probes that can easily identify resistant clones of high-risk in case of nosocomial infections or community outbreaks, e.g., Xpert MRSA/SA PCR Assay, Xpert vanA/vanB PCR Assay for VRE, Whole Genome Sequencing (WGS) for MDR-TB (WGS probes have been used to sequence the genomes of *Mycobacterium tuberculosis* strains, identifying mutations in the *katG* and *inhA* genes associated with isoniazid resistance), Fluorescent *In Situ* Hybridization (FISH) for ESKAPE Pathogens, Peptide nucleic acid FISH (PNA FISH) probes have been developed to identify high-risk clones of ESKAPE pathogens (Holzknecht et al., 2017; Tamilzhalagan et al., 2021; Yamada et al., 2023; Duan and Wang, 2022). Whole genome sequencing, one of the most convenient approaches of genomics can play a key role in managing the problem of antibiotic resistance. It can be helpful in quickly characterizing the pathogen, controlling its dissemination, choosing the effective drug, and recognizing the mechanism of resistance. A comparative analysis of the genome of *S. aureus* strains that are resistant to vancomycin (vancomycin-intermediate), *S. aureus* (VISA-MIC between 4 and 8 mg/L) or heterogeneous VISA (hVISA-strains appear to be sensitive to vancomycin with susceptible range of 1–2 mg/L, but containing subpopulation of vancomycin-intermediate daughter cells (MIC ≥4 μg/mL)(CLSI)) and their vancomycin-sensitive parent strain disclosed the role of mutation in resistance. Mutation in genes that encode *vraSR* and *graSR* the component regulatory system was the cause of resistance in these strains (Matsuo et al., 2013; Kim and Lee, 2020). Another example of how genomic studies help in tracking resistance genes is the discovery of the contribution of the *mfd* gene (responsible for transcription repair coupling factor) in *Campylobacter jejuni*. Transcription profiling of fluoroquinolone resistance in *Campylobacter jejuni* after the administration of ciprofloxacin showed up-regulation of the *mfd* gene. Mutation in the mfd gene led to a decrease in spontaneous mutation to ciprofloxacin resistance. Moreover, the loss of the *mfd* gene resulted in reduced resistance against fluoroquinolone outstandingly. The *mfd* protein increases the mutation rate by promoting error-prone DNA repair mechanisms when antibiotics like ciprofloxacin damage the bacterial DNA. This results in the emergence of mutations in the genes encoding DNA gyrase and topoisomerase IV, which reduces the binding affinity of fluoroquinolones and leads to resistance. A significant finding is that bacteria lacking the mfd gene show a reduced rate of spontaneous mutations to ciprofloxacin resistance. This suggests that *mfd* is directly involved in facilitating the mutations that confer resistance. Deletion of the *mfd* gene has been shown to reduce the resistance level in bacterial populations, e.g., in *E. coli*, researchers observed that *mfd* gene deletion resulted in a significant decrease in the frequency of mutations which conferred resistance to ciprofloxacin. The study further demonstrated that the absence of *mfd* impaired the bacteria’s ability to survive fluoroquinolone treatment, leading to a lower incidence of resistant colonies (Strick and Portman, 2019).

### 3.6 Awareness among the general public and antibiotic resistance stewardship programs

The development of antibiotic resistance in both pathogenic and non-pathogenic microbes is multifaceted. Dissemination of antibiotic resistance is not only limited to the clinical use of antibiotics but is also affected by their non-clinical usage. For decades antibiotics have been in use as a preventive measure in various fields like agriculture, aquaculture, animal husbandry, etc. Moreover, the use of antibiotics as growth promoters or supplements for livestock is also a factor responsible for its spread. Foodborne illness and bad hygiene also result in the frequent occurrence of bacterial infections, thus playing a direct role in the dissemination of antibiotics and subsequently antibiotic resistance (Uddin et al., 2021). Irrational use of antibiotics and their disposal in household waste are a major social contributor to antibiotic resistance dissemination (Sweileh, 2021; Londong et al., 2023). One of the many reasons behind the irrational use of antibiotics is a lack of awareness and knowledge on antibiotic use and antibiotic resistance. The irrelevant use and consumption of antibiotics, their over-the-counter availability in low, lower-middle, upper-middle-income countries, and the absence of knowledge regarding the safe disposal of expired and unused antibiotics have increased antibiotic resistance by manifolds (Voicu et al., 2021). Tackling antibiotic resistance by developing new antibiotics and other strategies alone is cumbersome (Pokharel et al., 2019; Godman et al., 2021). The phrase “Prevention is better than cure” stands correct in case of increasingly developing antibiotic resistance globally. Educating people about good hygiene and food practices to reduce infection rates, antibiotics, rational use of antibiotics and antibiotic resistance will help in controlling and slowing the spread and evolution of resistance to some extent (Ha et al., 2019; Godman et al., 2021; Maillard et al., 2020). For example, In France, a seasonal public health campaign to spread public awareness of viral respiratory infections and antibiotic resistance led to a 27% reduction in antibiotic use over 5 years (WHO, 2018). The Fourth joint inter-agency report on integrated analysis of antimicrobial consumption and occurrence of antimicrobial resistance in bacteria from humans and food-producing animals in the European Union (JIACRA IV-2019-2021) supports the fact that reducing the antimicrobial consumption helps in lowering overall antibiotic resistance. This report also highlights the importance of promoting measures such as vaccination and better hygiene in both human and animal health to reduce the need for antimicrobials. Since rational use of antibiotics requires multiple interventions in a comprehensive manner, not only through citizen campaigning but also through improving health workers’ prescribing and dispensing competency, WHO along with its partners has launched The Antibiotic Resistance Stewardship Program. Such programs are a step forward in making people aware of the significant challenges of antibiotic resistance and helping them use antibiotics rationally. In the United Kingdom, the *“Keep Antibiotics Working”* campaign was launched in 2017 by Public Health England (PHE), which helped achieve a significant reduction in antibiotic prescriptions. Between 2015 and 2019, general practitioners in the UK reduced antibiotic prescriptions by 17%, contributing to an overall 7% reduction in antibiotic use across healthcare settings (Public Health England, 2020). Another example of an awareness campaign is the *“Antibiotics Off the Menu”* initiative by the Natural Resources Defense Council (NRDC) in the United States, aimed at reducing antibiotic use in the food industry, successfully influenced large fast-food chains such as McDonald’s, KFC, and Subway to limit the use of medically important antibiotics in their meat production. Following these commitments, McDonald’s reported an 85% reduction in medically important antibiotics in its U.S. chicken supply chain by 2018 (NRDC, 2021).

In India, the *“MedWatch”* initiative by the Indian Council of Medical Research (ICMR), launched in 2018, has focused on monitoring antibiotic prescriptions and raising awareness in hospitals. This program, combined with public awareness efforts, contributed to a 20% reduction in antibiotic misuse in targeted hospitals by 2020 (ICMR, 2020). The finding of the survey study conducted by Mittal et al. in 2023 strengthened the case for regular conduct of continuing medical education and training of clinicians on various aspects related to antimicrobial resistance, surveillance, and use. The study identifies certain areas deserving of attention due to knowledge gaps. A few areas deserving attention are antibiograms along with their interpretation and applicability of appropriate agent selection, WHO AWaRe classification of antibiotics, guideline-based recommendations for optimal use of antibiotic agents and duration of antibiotic therapy, double anaerobic cover and double cover for gram-negative infections, irrational antibiotic combinations, and intravenous to oral switch of antibiotics when clinically desirable. Besides these, there is a need to emphasize the crucial role of infection prevention and control measures, including hand hygiene, not only among healthcare professionals but the community as a whole (Mittal et al., 2023). Despite these efforts, studies show that in low-and middle-income countries, irrational use of antibiotics remains high underlining the need for broader education and stricter regulation (Kanan et al., 2023). The continued promotion of responsible antibiotic use through public campaigns, coupled with policies aimed at improving health workers’ prescribing practices, is crucial in addressing the rising threat of antibiotic resistance. These initiatives show that public education campaigns, when paired with regulatory and healthcare interventions, can significantly reduce antibiotic misuse. Increasing awareness of AMR and sensitization and training of clinicians on various listed issues is a key strategy for safe and rational use of antimicrobials and heralds the menace of AMR at local, national, as well as global, levels.

## 4 Conclusion

Antibiotics have saved the human community from life-threatening bacterial diseases. Still, their exploitation or overuse has caused the emergence of resistant strains due to which antibiotic treatment has become ineffective. The anticipation that the thoughtless use of antibiotics could result in the spread of resistant strains of bacteria has become a reality. Today overcoming antibiotic resistance requires multiple efforts and strategic use of currently available antibiotics. Accurate and specific diagnosis of bacterial infections is paramount in reducing antibiotic resistance. Identifying the exact pathogen responsible for an infection allows for the targeted use of antibiotics, minimizing the use of broad-spectrum agents that can contribute to the emergence of resistant strains. By employing advanced diagnostic tools, such as molecular assays and next-generation sequencing, healthcare providers can ensure that the most effective and appropriate antibiotic is prescribed. This approach not only enhances treatment efficacy but also curtails the inadvertent promotion of resistance. The role of appropriate antibiotic prescribing in mitigating resistance cannot be overstated. Correct antibiotic use involves selecting the right drug, dosage, and duration tailored to the specific infection. Adhering to established guidelines and stewardship programs helps prevent over-prescription and misuse, which are significant drivers of antibiotic resistance. Educating healthcare providers on the principles of antibiotic stewardship and ensuring adherence to best practices are essential steps in reducing the spread of resistance. Furthermore, patient education on the importance of completing prescribed courses and not self-medicating with antibiotics is crucial in supporting these efforts. Strategies such as Host-Directed Therapy, Stem Cells, Antibacterial Viral Therapy can also help in combating antibiotic resistance. Host-directed therapies aim to enhance the body’s defense mechanisms against infections. These therapies include immunomodulators and agents that boost the host’s immune response, potentially reducing reliance on antibiotics and minimizing the risk of resistance. By targeting the host’s immune system, these therapies can complement antibiotic treatments and contribute to more effective infection management. Stem cell therapy holds promise in addressing antibiotic resistance by promoting tissue repair and regeneration. Infected or damaged tissues often require prolonged antibiotic treatment, which can contribute to resistance. Stem cells may offer an alternative by aiding in the healing process and potentially reducing the duration of antibiotic use. Research into the efficacy and safety of stem cell therapies in infection management is ongoing, with the potential to offer significant benefits. Bacteriophages and other viral-based therapies present a novel approach to targeting bacterial infections. These therapies are designed to specifically infect and kill bacterial cells, offering a targeted alternative to traditional antibiotics. The use of bacteriophages, in particular, has shown promise in treating multidrug-resistant infections, and ongoing research aims to optimize their effectiveness and safety in clinical settings (van den Biggelaar et al., 2024; Bergman et al., 2020; Yoshitani et al., 2020; Ling et al., 2022). Utilization of combination therapies and antibiotic adjuvants has been proven to be a promising approach for fighting against this resistance challenge. Some successful examples of combinational therapy treatments are combination of Trimethoprim and Sulfamethoxazole to treat a variety of infections, including urinary tract infections, respiratory infections, and *Pneumocystis jirovecii* pneumonia; Isoniazid, Rifampicin, Pyrazinamide, and Ethambutol combination to treat Tuberculosis (World Health Organization, 2010). Treatment of tuberculosis: guidelines, antibiotic combinations such as Clarithromycin, Amoxicillin (or Metronidazole), and a Proton Pump Inhibitor (PPI) like Omeprazole to treat *H. pylori,* etc. However, Resistance to combination therapies, once considered a robust strategy against antibiotic-resistant infections, is indeed on the rise, raising concerns about the future effectiveness of these treatments. Development of cross-resistance, compensatory mutations, biofilm formation, intrinsic resistance, inadequate dosing or inconsistent use, etc. resulted in the development of resistance against combinational therapies too. Moreover, the effectiveness of such combinations varies widely depending on the pathogen and the resistance mechanisms involved. Optimizing these combinations to minimize side effects and prevent resistance development requires further research. Clinical trials are needed to identify the most effective pairings and ensure their safety and efficacy. The use of inhibitors against enzymes responsible for resistance to antibiotics is a highly successful scheme such as BLIs and inhibitors of aminoglycoside modifying enzymes. Various inhibitors of efflux pumps have been used to inhibit certain efflux pumps and therefore, prevent the expulsion of antibiotics out of bacterial cells. Expanding the range of enzyme inhibitors to cover other resistance mechanisms and ensuring their stability and effectiveness in clinical settings is essential. The development of new inhibitors tailored to emerging resistance enzymes is critical for staying ahead of resistant strains. Targeting biofilm formation and quorum sensing can help in obstructing antibiotic resistance and this can be achieved using various quorum quenchers and inhibitors of biofilm formation. Alterations or modifications in the outer membrane of bacteria cells enhance the uptake of antibiotics. This can be achieved by using several membrane permeabilizers that increase the permeability of the outer lipid bilayer in bacteria. Delivery of antibiotics by loading them on organic and inorganic nanoparticles has turned out to be a useful technique to increase both their efficacy and movement across the membrane. All these approaches and strategies of using genomics and anti-plasmid methods and their developing success against antibiotic resistance give hope of getting better control over the emerging threat of antibiotic resistance. These approaches also proved advantageous in understanding the mechanisms involved in the development and dissemination of antibiotic resistance. Other than this, certain natural products, including essential oils, have demonstrated antimicrobial properties and may serve as adjunctive treatments in combating resistant infections. Essential oils such as tea tree oil, oregano oil, and eucalyptus oil possess antibacterial and antifungal activities that could complement conventional antibiotic therapies. Their use in infection management, combined with their potential to reduce antibiotic dependence, makes them a valuable area of exploration. In conclusion, addressing antibiotic resistance requires a multifaceted approach that includes precise diagnosis, appropriate antibiotic use, and the exploration of innovative treatment strategies. By integrating these measures, we can improve infection management and reduce the impact of antibiotic resistance on public health. Development and optimization of these approaches to make them more accessible and effective will help in tackling the problem of antibiotic resistance more urgently and with the greatest degree of precision and provide the time necessary for the development of new and better antibiotics, which is the need of the hour.

## Footnotes

^1^https://pubmed.ncbi.nlm.nih.gov/21793988/
